# Kin Discrimination Modifies Strain Distribution, Spatial Segregation, and Incorporation of Extracellular Matrix Polysaccharide Mutants of Bacillus subtilis Strains into Mixed Floating Biofilms

**DOI:** 10.1128/aem.00871-22

**Published:** 2022-09-12

**Authors:** Maja Bolješić, Barbara Kraigher, Iztok Dogsa, Barbara Jerič Kokelj, Ines Mandic-Mulec

**Affiliations:** a University of Ljubljana, Biotechnical Faculty, Department of Microbiology, Ljubljana, Slovenia; b University of Ljubljana, Chair of Microprocess Engineering and Technology – COMPETE, Ljubljana, Slovenia; University of Michigan-Ann Arbor

**Keywords:** *Bacillus subtilis*, biofilms, kin discrimination, incorporation of extracellular matrix polysaccharide mutant

## Abstract

Microorganisms in nature form multicellular groups called biofilms. In biofilms, bacteria embedded in the extracellular matrix (ECM) interact intensely due to their proximity. Most studies have investigated genetically homogeneous biofilms, leaving a gap in knowledge on genetically heterogeneous biofilms. Recent insights show that a Gram-positive model bacterium, Bacillus subtilis, discriminates between strains of high (kin) and low (nonkin) genetic similarity, reflected in merging (kin) and boundaries (nonkin) between swarms. However, it is unclear how kinship between interacting strains affects their fitness, the genotype assortment, and incorporation of the mutant lacking the main structural ECM polysaccharide (EpsA-O) into floating biofilms (pellicles). We cultivated Bacillus subtilis strains as mixtures of isogenic, kin, and nonkin strain combinations in the biofilm-promoting minimal medium under static conditions, allowing them to form pellicles. We show that in nonkin pellicles, the dominant strain strongly reduced the frequency of the other strain. Segregation of nonkin mixtures in pellicles increased and invasion of nonkin EpsA-O-deficient mutants into pellicles decreased compared to kin and isogenic floating biofilms. Kin and isogenic strains had comparable relative frequencies in pellicles and showed more homogenous cell mixing. Overall, our results emphasize kin discrimination as a social behavior that shapes strain distribution, spatial segregation, and ECM mutant ability to incorporate into genetically heterogenous biofilms of B. subtilis.

**IMPORTANCE** Biofilm communities have beneficial and harmful effects on human societies in natural, medical, and industrial environments. Bacillus subtilis is a biotechnologically important bacterium that serves as a model for studying biofilms. Recent studies have shown that this species engages in kin discriminatory behavior during swarming, which may have implications for community assembly, thus being of fundamental importance. Effects of kin discrimination on fitness, genotype segregation, and success of extracellular matrix (ECM) polysaccharide (EpsA-O) mutant invasion into biofilms are not well understood. We provide evidence that kin discrimination depends on the antagonism of the dominant strain against nonkin by using environmental strains with determined kin types and integrated fluorescent reporters. Moreover, this antagonism has important implications for genotype segregation and for when the bacteria are mixed with ECM producers. The work advances the understanding of kin-discrimination-dependent bacterial sociality in biofilms and its role in the assembly of multicellular groups.

## INTRODUCTION

Biofilms are multicellular assemblages of bacteria that engage in intense social interactions that bring about positive (cooperative) or negative (antagonistic) fitness effects (1). Biofilms in natural habitats are the most dominant forms of microbial life and are predominantly composed of genetically diverse microorganisms (2–4). Nevertheless, most studies have focused on genetically homogenous biofilms.

The hallmark of biofilms is a matrix of self-generated extracellular polymeric substances (EPS) that glue cells together, mediate surface attachment, provide stability, and improve survival in harsh environments (5). Extracellular matrix (ECM) polymers serve as “public goods” because they are released and utilized by cells joined in biofilm collectives (6). However, we have limited understanding of ECM exploitation in genetically heterogeneous biofilms. According to the work of Hamilton (7, 8), organisms apply kin discrimination to match their behaviors toward others according to their genetic similarity. Moreover, kin discrimination may limit the possibility of less similar genotypes exploiting costly public goods during group living, due to cells’ sorting, avoidance, or antagonism between nonkin (9, 10).

Studies on bacterial kin discrimination have mostly employed cooperative swarming as the test behavior to determine phenotypic responses of bacterial strains at the point of encounter, where a visible boundary between two strains was found to indicate nonkin interactions. In contrast, merging was found to be more common between swarms of very close kinship or between isogenic swarms (11–16). Recently, we showed that the nonpathogenic, spore-forming, Gram-positive soil bacterium Bacillus subtilis engages in kin discrimination during swarming, where the frequency of the boundary appearance or merging at the meeting point of two swarms correlates with genetic similarity. Genetic similarity between interacting strains was defined by comparing the identities of four housekeeping genes (13) and by average nucleotide identity (ANI) (17, 18) of orthologous gene pairs shared between two microbial genomes. Specifically, B. subtilis kin strains with 99.93 to 99.99% ANI and isogenic strains (“self” pairings, 100% ANI) exhibited merging behavior, whereas less genetically similar strains (98.73 to 98.84% ANI), which we also refer to as nonkin strains, formed clear boundaries (18). Moreover, Lyons et al. showed that less genetically similar B. subtilis strains also differ in a combination of kin discrimination loci, which comprise genes for antimicrobials, contact-dependent toxin-immunity pairs, and the enzymes involved in the synthesis of major ECM polysaccharide EpsA-O (16). However, we lack information on how kin discrimination affects fitness and cell sorting of different genotypes in biofilms (19).

B. subtilis is a model organism that is often used to investigate biofilm development on plant roots (13, 20, 21), on agar surfaces (22), or at the air-liquid (A-L) interface, where B. subtilis constructs intricate pellicles (23–26). Pellicle formation is dependent on biofilm ECM polysaccharide EpsA-O and the TasA amyloid protein fibers, anchored through TapA to peptidoglycan (24, 27–29). B. subtilis ECM mutants form poor pellicles alone, but coculturing of the *tasA* and *epsA*-*epsO* operon (Δ*epsA–O*) mutants that are isogenic in other loci compensates for the defect in pellicle formation (27, 28, 30, 31). It is not known whether Δ*epsA–O* mutants can incorporate into pellicles when mixed with the phylogenetically more distant (nonkin) ECM-positive strains.

We here test the hypothesis that low kinship will induce spatial segregation and will change the relative cell frequency of interacting B. subtilis strains in genetically mixed biofilms. Moreover, we predict that nonkin interactions between B. subtilis strains will limit the invasion of the Δ*epsA–O* mutant into the pellicle when cocultured with an ECM producer. To test these predictions, we use selected natural B. subtilis strains isolated from one 1-cm^3^ soil sample (32) with previously determined genetic similarity and kin discrimination phenotype (13, 16, 18).

We show that two nonkin B. subtilis strains behave differently from kin or isogenic mixtures in pellicles. In nonkin pellicles, the dominant strain decreases the fitness of the partner strain, whereas this dominance is not detected in kin and isogenic pellicles. Also, in nonkin pellicles, strain segregation is more prominent than in kin and isogenic pellicles, where two strains remain more intermixed. Moreover, nonkin interactions also restrict the incorporation of the Δ*epsA–O* mutant into pellicle when mixed with the ECM producer. The findings of this work make an important contribution to our understanding of how kin discrimination shapes bacterial biofilms, which is relevant for biofilm control and application.

## RESULTS

### Kinship-dependent social interactions reveal dominance of one strain in Bacillus subtilis mixed floating biofilms.

**FIG 1:**
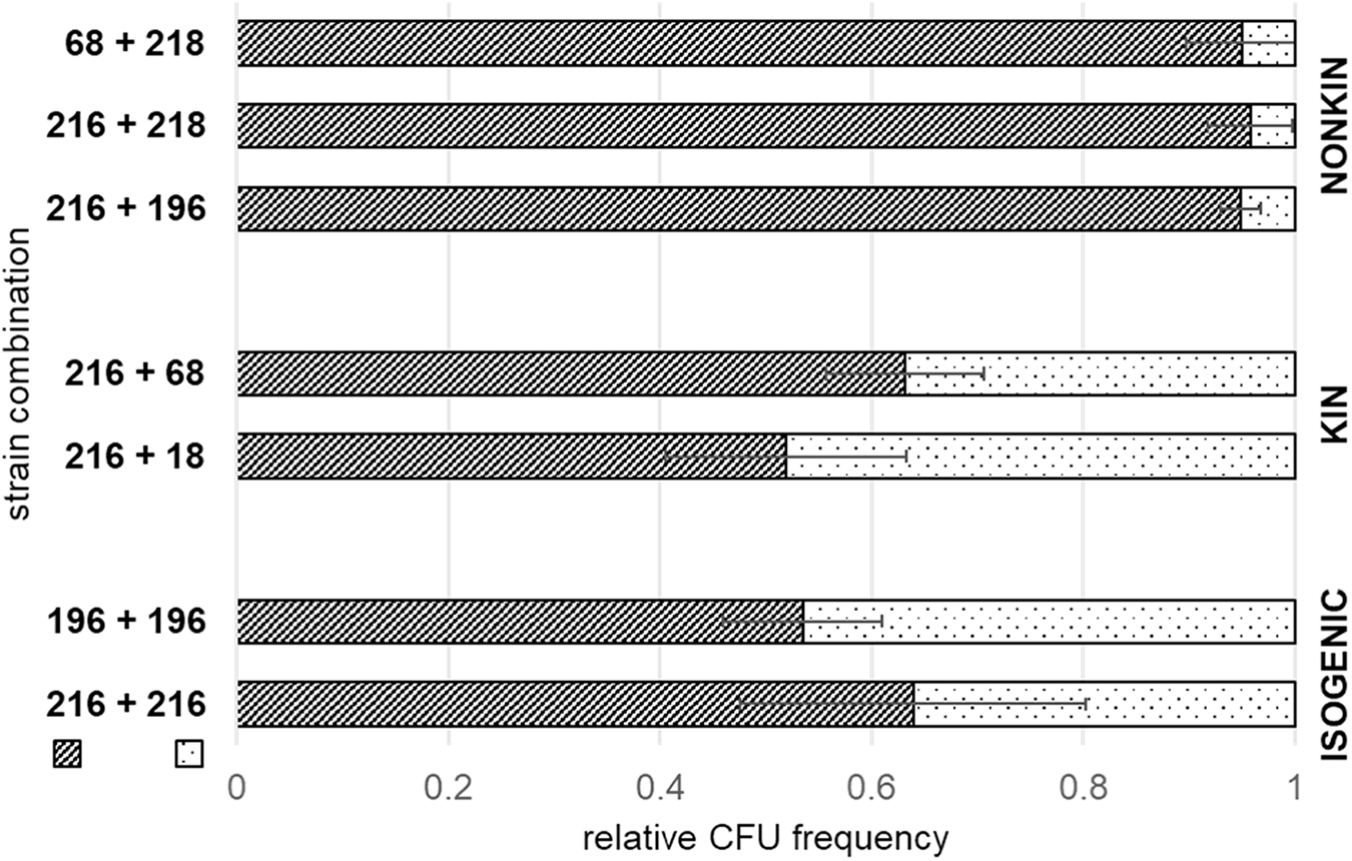
Relative cell frequency of two strains in mixed pellicles is influenced by nonkin interactions. The relative cell frequencies in pellicles of two isogenic (the same strain labeled with different fluorescent markers), two kin, and three nonkin strain combinations are shown. The first strain in the combination is always represented by the darker bar with the diagonal pattern. Two strains differentially labeled with different antibiotic resistance markers were mixed as revitalized spores in given combinations (designated numbers of PS strains). They were grown in 2 mL of MSgg for 24 h at 37°C, and the relative frequency of each strain was assessed in the formed pellicles after sonication by CFU counts. The proportions of the two strains in the pellicles were significantly different in kin and isogenic strain combinations than in nonkin strain combinations (Student *t* test; two-tail, *P* < 0.05). Mean values of relative frequency of the first strain in each strain combination (±SD) are shown as darker bars with a diagonal pattern. Experiments were performed in at least three biological replicates (*n* ≥ 3).

Bacteria in natural settings reside in genetically mixed collectives. To monitor the distribution of different strains in mixed floating biofilms, we used strains which originate from a microscale soil environment (32). To provide the means to determine the cell numbers of each strain in pellicle competition assay by counting cells (CFU) on selective media, we labeled the strains used by constitutively expressed fluorescent reporters (yellow fluorescent protein [YFP] or red fluorescent protein [mKate2]) and associated antibiotic resistance (spectinomycin for strains marked with YFP and chloramphenicol for strains marked with mKate2). Two strains were inoculated together at a 1:1 ratio (10^5^ spores/mL each) in the biofilm-promoting minimal medium (MSgg) and then incubated under static conditions for 24 h. Table 1 shows the genetic similarity between the selected strains based on the mean nucleotide identity of orthologous gene pairs shared between two microbial genomes (ANI) (18). The results showed that one strain gained a fitness advantage over the other in nonkin pellicles, whereas in kin and isogenic pellicles the two highly genetically similar strains reached similar final cell counts. As shown in Fig. 1, in kin and isogenic strain combinations labeled with different fluorescent markers the relative frequency of each strain was close to its initial frequency (0.5). In contrast, in nonkin pellicles the final relative frequency of the two strains was significantly changed (Student *t* test; two-tail, *P* < 0.05). Mixing of two isogenic strains in the pellicle was tested for all five strains used in this study and indicated that the type of fluorescent reporter did not significantly affect the mixing of the strains in the formed pellicle (see Fig. S2 in the supplemental material). Next, we tested the influence of the physiological state of the cells used for the initial mix on the outcome in the biofilm competition assay. Results showed that exclusion between nonkin strains was stronger when using exponential-growth-phase cells for inoculum mix than when using revitalized spores. The dominant strains in the exponential-phase competition mixture represented at least 99.94% (Fig. S3). In contrast, when the revitalized spores were mixed, the proportion of the dominant strain was approximately 95% of the total CFU counts in the pellicle (Fig. 1). Furthermore, we compared the average growth rates of floating biofilms of the strains used. Although the growth curves showed slight differences between strains (Fig. S1), they did not affect the competition outcome. For example, the fastest-growing strain, PS-196 (Fig. S1), was outcompeted by strains that grew slower (PS-216, PS-218, and even the slowest-growing strain, PS-68) (Fig. 1 and Fig. S3). Altogether, our results are consistent with the prediction that kin discrimination-associated interactions lead to differential behavior of strains in nonkin biofilms, with one strain being dominant over the other.

**TABLE 1:** Average nucleotide identity values between strain pairs^*a*^

Kin group^*b*^	Strain	ANI value for strain:				
		PS-216	PS-18	PS-68	PS-196	PS-218
9	PS-216	*1.0000*				
9	PS-18	*0.9994*	*1.0000*			
9	PS-68	*0.9995*	*0.9993*	*1.0000*		
3	PS-196	**0.9883**	**0.9881**	**0.9884**	*1.0000*	
7	PS-218	**0.9875**	**0.9875**	**0.9875**	**0.9877**	*1.0000*

a Values were retrieved from the work of Stefanic et al. (18). Boldface denotes nonkin combinations, and italic denotes kin combinations.

b Kin groups are numbered according to the work of Stefanic et al. (13).

### Kin discrimination affects spatial assortment of strains in floating biofilms.

**FIG 2:**
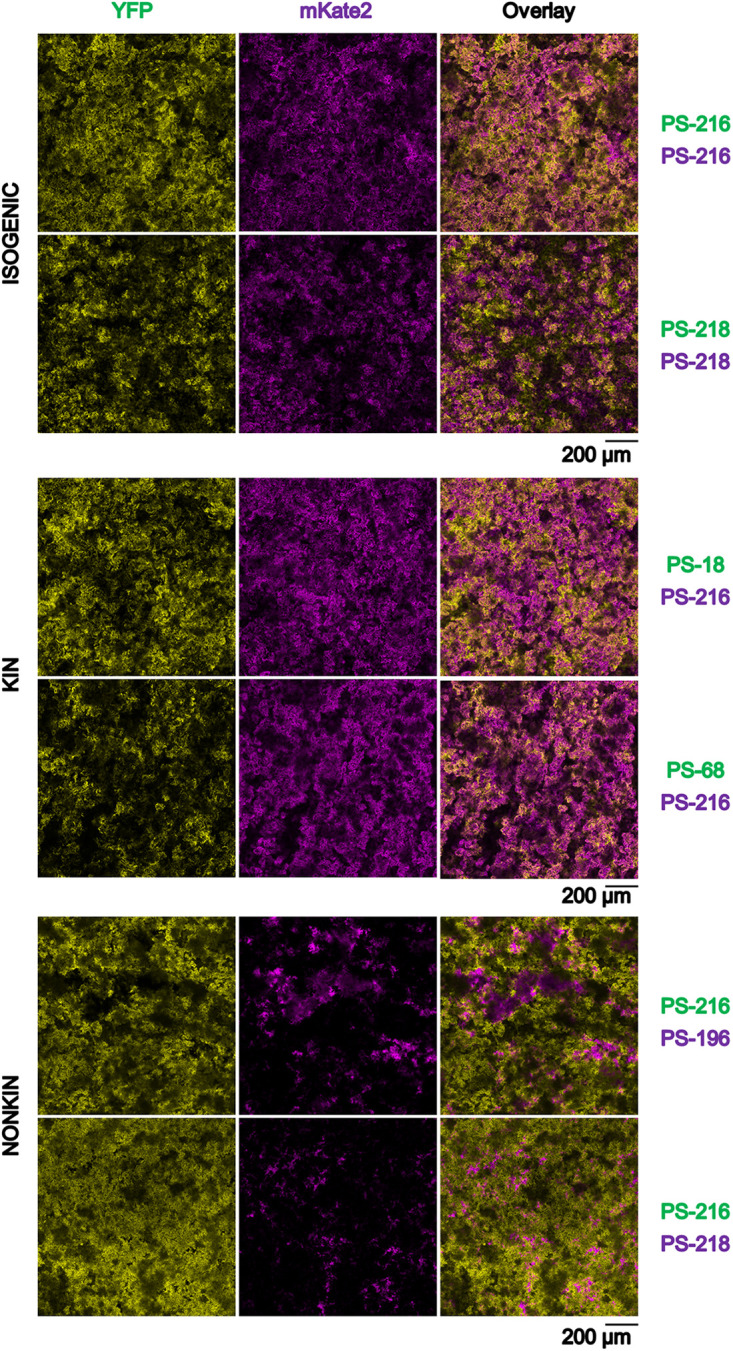
Fluorescent microscopy imaging of isogenic, kin, and nonkin strains in floating biofilms. Pellicles were formed by mixing of two isogenic, kin, and nonkin B. subtilis PS strains that constitutively express fluorescent reporters. Biofilms were examined by confocal laser scanning microscopy (CLSM) (bar, 200 μm) after 16 h of biofilm growth. This time point was chosen to ensure imaging before sporulation started. Images were pseudocolored yellow (for YFP-tagged B. subtilis strains PS-216, PS-218, PS-18, and PS-68) and magenta (for mKate2-tagged strains B. subtilis PS-216, PS-218, and PS-196). Representative images of at least three biological replicates are shown.

**FIG 3:**
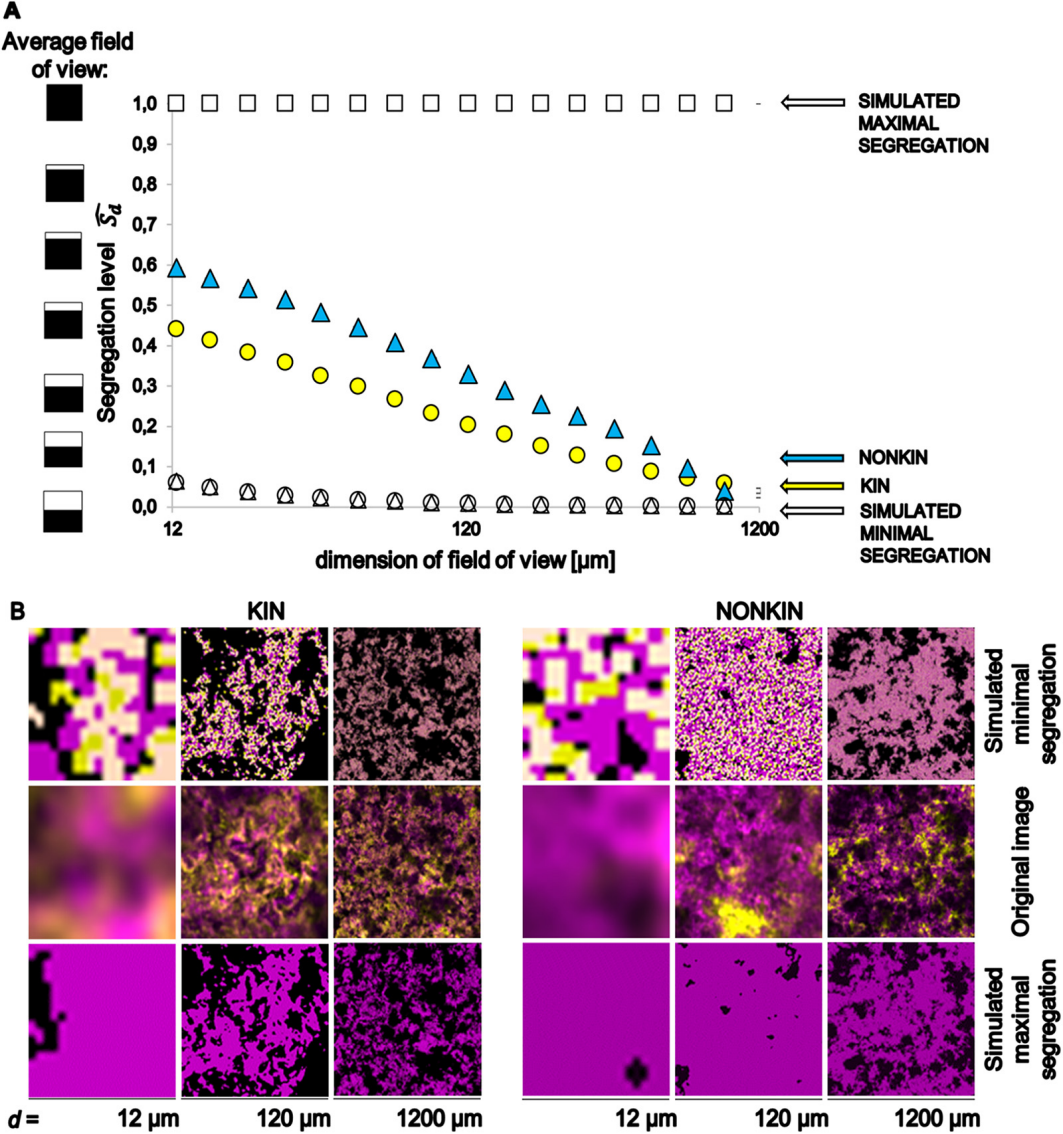
Multiscale spatial segregation analysis (MSSA) of the floating biofilm’s image stacks obtained by confocal laser scanning fluorescence microscopy (CLSM). (A) Plots of calculated segregation levels ($\hat{S_{d}}$) in mixed strain biofilms are shown for the kin pair (PS-68 YFP + PS-216 mKate2) strains and nonkin pair (PS-218 YFP + PS-216 mKate2) strains as a function of the dimension of field of view. Examined field of view, which represents what is seen under the microscope at different zoom levels, is a square with the dimensions *d* by *d*. $\hat{S_{d}}$ was calculated for simulated maximal and minimal segregations. $\hat{S_{d}}$ is 1 if only one strain is present in the field of view and 0 if both strains are present in the expected ratios (ratio of the two strains in all images of the stack) in the field of view, graphically represented as the average field of view. (B) Microscopy images (middle slice of CLSM stack) representing the field of view with *d* of 12 μm, 120 μm, and 1,200 μm. Top and bottom rows show corresponding simulated images; middle row represents original images as taken by the microscope. For the simulation of maximal segregation, it was assumed that one strain completely excluded the other strain.

Given that during pellicle formation B. subtilis strains engage in interactions with differential fitness effects for isogenic, kin, and nonkin pairs, we predicted that these interactions may also impact their spatial distribution in pellicles. To test this prediction, we grew mixed cultures as described above using the same pairs of strains labeled with two different fluorescent markers (YFP and mKate2). We examined pellicles by confocal laser scanning microscopy (CLSM) after removing the liquid medium. By visually inspecting slices of confocal images of biofilm stacks, it appeared that isogenic and kin cells were more homogenously dispersed, whereas in nonkin pellicles one strain occupied a larger surface/area than the other and segregation seemed more pronounced (Fig. 2 and Fig. S4). However, a biofilm confocal image comprised 15 to 25 image slices and the cell distribution varied from slice to slice. Hence, the intensity of the fluorescent marker may have influenced our visual perception of the strain distribution in the images, making it challenging to obtain an accurate estimation of overall segregation. In addition, the spatial segregation can be dependent on spatial scale, i.e., what appears to be well mixed when observed from a great distance can appear strongly segregated when observed from close proximity. Therefore, we applied an objective estimator of cell segregation that considers spatial scale of segregation. Specifically, we recently developed multiscale spatial segregation analysis (MSSA) applicable to the CLSM image biofilm’s stacks (detailed information available in reference 33). Briefly, the spatial scale is represented by different sizes of the field of view, which is a square with dimensions *d* by *d*, with *d* ranging in our case from 12 μm to 1.2 mm, and represents the observable area in a microscopy image (Fig. 3A). When field of view is 1.2 mm, the segregation is low. As we zoom in, and thereby decrease the size of the field of view, the segregation becomes apparent and more intense in nonkin pellicles (Fig. 3A and B). For the experimental pellicle in the field of view of 12 μm by 12 μm, the segregation level ($\hat{S_{d}}$) was 0.44 for the kin pellicle and 0.6 for the nonkin pellicle (Fig. 3A). Accordingly, the segregation level expressed in terms of the strain ratios in the field of view (see equation 2) reveals the segregation effect to be more pronounced in nonkin pairs (7:3 for kin versus 12:3 for nonkin pair). For the calculation of the maximal segregation level $\left(\hat{\;S_{d}^{\max}}\right)$, it was assumed that one strain completely excluded the other strain and that thus only this strain is present in each image slice. This implies that in this case one strain has completely eliminated the other strain. To simplify the comparison of several segregation-level curves and several combinations of strains depicted in Fig. S5, we calculated the relative multiscale spatial segregation level (rMSSL), which is $\hat{S_{d}}\;$ (adjusted for $\;\hat{\;S_{d}^{\max}}$ and $\hat{S_{d}^{\min}}$) averaged over all field of view dimensions. The results are given in Table 2 and show that the rMSSL of isogenic and kin strains is the same (within experimental error) but significantly differs (Student *t* test; two-tail, *P* < 0.05) from the rMSSL of nonkin strains.

**TABLE 2:** Relative multiscale spatial segregation levels of isogenic, kin, and nonkin two-strain biofilms^*a*^

mKate2-labeled strain	rMSSL value for combination with YFP-labeled strain:				
	PS-18	PS-68	PS-196	PS-216	PS-218
PS-18	**0.1**				
PS-68		**0.12**			*0.2*
PS-196			**0.11**	*0.21*	
PS-216	0.12	0.09	*0.25*	**0.1**	*0.14*
PS-218		*0.17*		*0.21*	**0.1**

a Average and SD of rMSSL values for the particular type of strain combination show significant differences between isogenic/kin and nonkin strain combinations (Student *t* test; two-tail, *P *< 0.05). For isogenic (bold), kin (underlined), and nonkin (italic) combinations, the average (SD) values are 0.10 (0.01), 0.11 (0.02), and 0.21 (0.03), respectively. Higher rMSSL values indicate more intense segregation.

### Kin discrimination limits incorporation of the *epsA–O* mutants into the pellicle when mixed with the extracellular matrix producer.

ECM is considered a public good that is shared by neighboring cells but which could be exploited by “mutants” that benefit from the cooperation without paying the cost (1, 34). B. subtilis 3610 wild type (WT), which forms a robust pellicle, complements the ECM-deficient-mutants unable to form a pellicle alone (27, 28, 30, 31).

**FIG 4:**
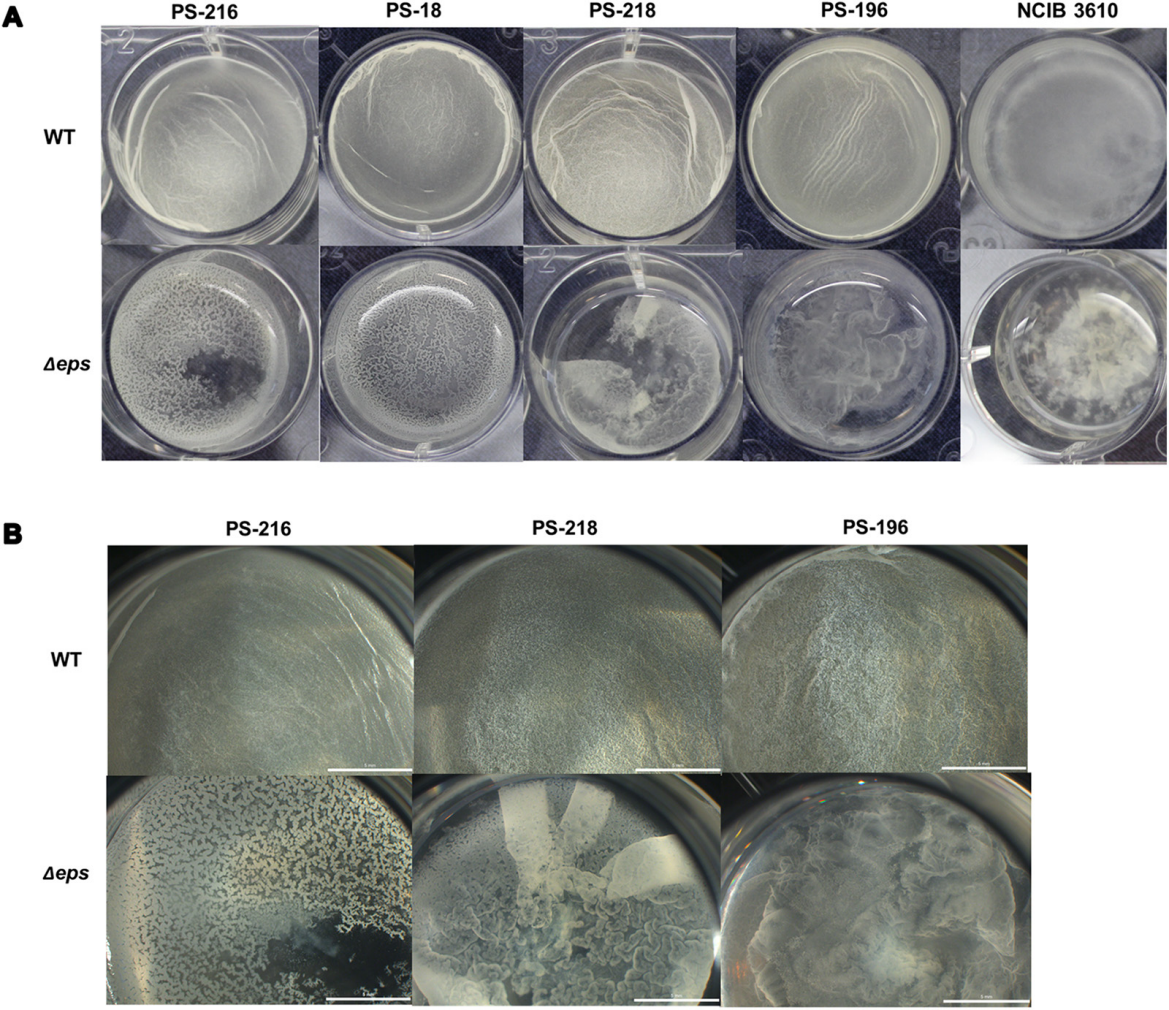
Effect of *epsA–O* mutation on floating biofilm formation in different B. subtilis strains. (A) Indicated strains were inoculated in exponential growth phase as monocultures in 12-well plates in 2 mL MSgg medium and incubated at 37°C. Images of representative biofilms in the wells (diameter, 20.5 mm) after 20 h of growth are shown. (B) Close-up images of pellicles of the indicated strains obtained by stereomicroscope; bar, 5 mm.

We first tested the effect of *epsA–O* inactivation on floating biofilm formation in different monoculture strains after 24 h of incubation at 37°C by inoculating the cells from exponential growth phase into 2 mL of MSgg medium in 12-well plates. The effect of *epsA–O* mutation on biofilm formation in different B. subtilis strains is shown in Fig. 4. The mutation induced a visible effect on pellicle formation in all the tested strains, although the morphology of the mutant biofilms differed between the strains (Fig. 4). Our most studied strain, PS-216, still formed floating aggregates at the A-L interface even with the deleted *epsA–O*, but the morphology was markedly changed compared to the WT (Fig. 4). The biofilms formed by the PS-216 Δ*epsA–O* strain appeared less thick, and the cell aggregates were only slightly glued together, which is consistent with recently published results (26). The mutant strain PS-18 (which is kin with PS-216) formed pellicles which were fragile and morphologically very similar to those of the PS-216 Δ*epsA–O* mutant, while the other tested strains formed submerged aggregates of cells, which were not reminiscent of pellicles at the A-L interphase (Fig. 4).

**FIG 5:**
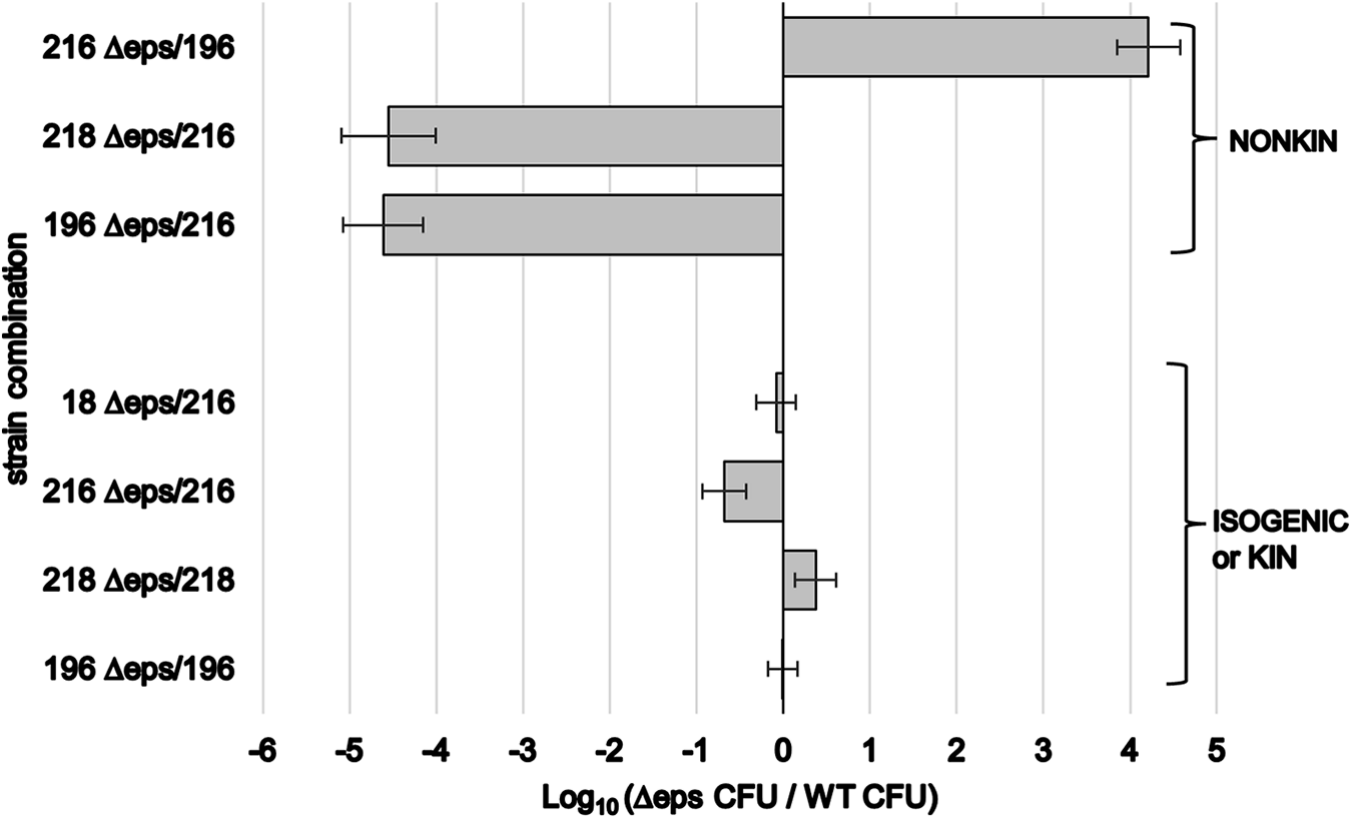
Incorporation of isogenic (or kin) Δ*epsA–O* mutant strain into the pellicle when mixed with the wild-type (WT) EpsA-O producer and exclusion of nonkin strains. Two strains differentially labeled with different antibiotic resistance markers were mixed in exponential growth phase in given combinations (numbers represent specific PS strain; Δ*eps* is an indication of the mutant). They were grown in 2 mL of MSgg for 24 h at 37°C. Log_10_ values of the final mean ratios between Δ*epsA–O* mutants and WTs (±SD) determined by CFU counts in the formed pellicles after sonication are shown. Experiments were performed in at least four biological replicates (*n* ≥ 4), except for the kin combination PS-18 Δ*epsA–O* mixed with PS-216 (*n* = 2).

Incorporation of ECM-deficient strains into the WT pellicle is enabled by exploitation of WT ECM components (27, 28, 30, 31). Thus, we examined whether strain genetic similarity will affect the success of the Δ*epsA–O* mutants in occupying floating biofilms. We predicted that incorporating the mutants into pellicles would be more efficient in isogenic/kin than in nonkin mixes. The Δ*epsA–O* mutants were mixed with the parental or nonkin WT strains (in exponential growth phase at a 1:1 starting ratio) in a 2-mL volume, and the incorporation of the mutants into the pellicle was monitored after 24 h of incubation at 37°C by CFU counts. Similar to the results obtained by mixing of the two WT strains, a strong exclusion of the nondominant strain was detected in nonkin mixes involving Δ*epsA–O* mutants. In nonkin mixes we detected up to a 4-log_10_ difference between the final mean ratios of the Δ*epsA–O* mutant and the WT cell counts in pellicles. In contrast, the final mean ratios of the two strains in pellicles in isogenic and kin mixes were more similar (Fig. 5). While isogenic mixtures mixed efficiently in pellicles, in nonkin mixes, the dominant PS-216 strain excluded the nonkin PS-196 strain regardless of whether PS-216 produced EpsA-O or not (Fig. 5). Similar results were revealed by visually observing the pellicle phenotype of the PS-218 Δ*epsA–O* mutant mixed with PS-196. The mutant excluded the EpsA-O producer and failed to form a mixed pellicle (Fig. S6).

To further verify these results and the effect of kin discrimination on incorporation efficiency, we also mixed (exponential-growth-phase cells at a 1:1 starting ratio) the Δ*epsA–O* mutants of the two selected strains PS-216 and PS-196 in isogenic or nonkin pairs in 50 mL medium in Falcon tubes with a loosely closed lid to increase an oxygen gradient. Consistent with the results in Fig. 5, the two mutants successfully invaded the pellicle in 50 mL medium when mixed with the parental strain. In contrast, in nonkin biofilms, the dominant strain (PS-216), even if deficient in EpsA-O production, outcompeted PS-196 with an approximately 4-log_10_ (CFU) advantage (Fig. S7), indicating that competition outcome between nonkin strains is not dependent on the strain’s ability to produce ECM. One of the explanations for this result could be that the PS-216 Δ*epsA–O* mutant still forms fragile biofilms alone (Fig. 4); hence, it does not gain an advantage by exploiting ECM of PS-196 WT. The other is that PS-216 antagonism directs the competition and exclusion from the pellicle does not depend on the ECM production trait.

Overall, these results suggest that EpsA-O-deficient mutants efficiently incorporate into the floating biofilms when combined with isogenic WT strains, which is consistent with previous results (27, 28, 30, 31), but in nonkin interactions the formation of the mixed pellicles is restricted.

## DISCUSSION

Biofilms are composed of ECM-embedded bacteria that are capable of various social interactions that might affect fitness, cell assortment, and ECM exploitation and consequently structure and ecology of microbial communities (9). By using a subset of B. subtilis strains from micrometer soil aggregates (32) and with previously determined kin types (13), we here show strong interference competition between nonkin and the coexistence of kin and isogenic strains in pellicles. Nonkin strains also segregate into patches of different size and do not form well-mixed pellicles with EpsA-O-deficient strains as was shown for isogenic strains.

We find a significant reduction in relative CFU frequency of one strain in mixed biofilms composed of two nonkin strains, with one strain showing a significant dominance over the other. For example, PS-216 won over PS-196 or PS-218 (Fig. 1). In contrast, the relative frequency of both strains in kin and isogenic pellicles was preserved (Fig. 1) (or was much less changed than in nonkin strain combinations when exponential-phase cells were used as inoculum) (see Fig. S3 in the supplemental material). The competitive reduction of one strain in nonkin pellicles is most probably a consequence of antagonism between nonkin. Antagonism between nonkin has been observed at the meeting point of two nonkin swarms of B. subtilis (13, 16, 18) but was absent if kin swarms interacted. Antagonism was suggested also for nonkin Proteus mirabilis (35, 36) and Myxococcus xanthus (14, 37) interactions. Our results are also similar to those of previous studies on the consequences of mixing genetically divergent Pseudomonas aeruginosa strains in static cultures (38, 39). Although these authors did not specifically address kin discrimination, they reported antagonism between genetically different strains in biofilms. It is generally believed that clonemates cooperate whereas genetically different cell lineages of the same species compete (1, 40, 41). Competitive strategies between nonkin comprise contact-dependent killing (12, 16, 42) and local exchange of antagonistic molecules between swarms (16, 43). Although swarms represent a different type of collectives than pellicles, it is possible that nonkin deploy similar types of interactions during swarming and pellicle development, although there are also pronounced differences in the opportunities for social interactions. During a swarming encounter assay, cells in the swarm are initially surrounded by their kin and only at the point of encounter of two nonkin swarming cells do they engage in direct cell-cell contact. At this point they are exposed to limitations stipulated by higher cell density and nutrient/space limitation. During pellicle formation, nonkin first engage in interactions in the liquid medium, where they exist as planktonic cells, interacting through secreted and diffusible factors and even EPS that engage cells in a loose network (5). Several hours later, probably when they sense a limitation of oxygen, they invade the A-L interface, start forming patches, and engage in direct cell-cell interactions (26, 31).

Our results demonstrate that in pellicles bacterial cells segregate into visible patches similar to those that have previously been shown for colonies (44, 45). However, the assortment of nonkin cells results in more prominent patches of the dominant strain that are interspersed by smaller patches of the less competitive strain. This is different from cell organization in kin pellicles, where mixing is more pronounced and less heterogenous. This assortment is consistent with modeling experiments testing interstrain competition in spatially structured environments between a bacteriocin producer and nonproducer, which resulted in killing-driven assortment of two genotypes (46, 47).

In addition to social interactions, the biofilm heterogeneity is also shaped by steep vertical oxygen gradients that result from oxygen consumption by bacteria and slow oxygen diffusion into the medium (5, 48, 49). Pellicle construction is a strategy for effectively obtaining oxygen at the liquid surface (24, 25, 50). A possible explanation for the role of ECM in maintaining pellicles at the liquid surface is a decrease in the specific gravity or/and intrinsic hydrophobicity of cells glued by ECM, which counteracts selective forces faced by planktonic cells (50). We observed that the benefit of EpsA-O for occupying the A-L interphase was more important for the strains PS-196, PS-218, and NCIB 3610 than for PS-216 and PS-18, which even with inactivated *epsA–O* genes still formed weakly aggregated cells at the liquid surface (Fig. 4), a finding that is in accordance with observations by Krajnc et al. (2022), who investigated biofilm formation of the PS-216 Δ*epsA–O* mutant and its parental strain over time in MSgg medium and a setting similar to what we used (26). The results also imply that PS-216 may encode additional polysaccharides that compensate for the lack of EpsA-O in the mutant. In fact, natural isolates of B. subtilis often carry the intact copy of the *ypqP* (*spsM*) gene, encoding a sugar epimerase likely to be involved in polysaccharide synthesis (51), which is inactivated due to the integration of the SPβ prophage in the standard biofilm NCIB 3610 strain (52), which also in our setting did not form a pellicle, while the *spsM* gene is intact in the PS-216 genome.

Next, we studied incorporation of the EpsA-O-deficient strain into the pellicle at the air-liquid interface when mixed with the parental or nonkin strain. Confirming previous work performed with the NCIB 3610 strain (27, 28, 30, 31), all tested Δ*epsA–O* mutants were able to incorporate into the pellicle and utilize EpsA-O produced by their parental strains. Although the success of incorporation into the pellicle slightly differed between selected Δ*epsA–O* strains in isogenic combinations, the difference was small. In contrast, the incorporation efficiency of mixing with the nonkin EpsA-O producer was very low. Most importantly, the results revealed that the dominant strains in the selected strain combinations outcompeted the other strain even if they were EpsA-O deficient (Fig. 5 and Fig. S7), which resulted in more fragile or submerged biofilm of the mutant (Fig. S6). This result suggests that during pellicle formation the dominance of one strain over the other might be linked to antagonism against the less competitive strains regardless of the ECM production efficiency and that through this mechanism kin discrimination limits ECM exploitation between B. subtilis strains. However, antagonistic interactions might be only one of the mechanisms shaping nonkin interactions in bacteria, and our results do not exclude additional mechanisms that could contribute to kin discrimination outcomes. For example, bacteria may produce molecular determinants for kin recognition which ensure preferential grouping with kin strains that share these determinants.

In conclusion, our results support the hypothesis that B. subtilis strains harbor antagonistic mechanisms directed toward nonkin strains, which operate through inhibition of pellicle formation of one strain and a modified assortment of nonkin groups in the floating biofilm. These mechanisms may limit the invasion of the WT groups by nonkin ECM nonproducers and thereby biofilm ECM exploitation.

## MATERIALS AND METHODS

### Strains and media.

Strains used in this study are described in Table S1 in the supplemental material. Briefly, WT Bacillus subtilis strains were isolated from two samples of 1 cm^3^ of soil from the sandy bank of the Sava River in Slovenia previously (32). Recombinant strains carried reporters for YFP or mKate2 from constitutively expressed promoters. Strains were constructed previously (13, 53) using a standard protocol by transforming the chromosomal DNA from strain NCIB 3610 carrying the two constitutively expressed fluorescent promoters (16, 54) into the *amyE* locus by homologous recombination and selection for antibiotic resistance—chloramphenicol (Cm) at 5 μg/mL for strains tagged with mKate2 or spectinomycin (Sp) at 100 μg/mL for YFP tagging.

The B. subtilis EpsA-O-deficient strains were obtained via transformation of the strains PS-216 *amyE*::P*_tapA_-*yfp, PS-196 *amyE*::P*_tapA_-*yfp, PS-218 *amyE*::P*_tapA_-*yfp, and PS-18 *amyE*::P*_tapA_-*yfp (13) (for experiments in 2 mL of MSgg medium) and PS-216 P*_hyperspank_-*mKate2 (13) and PS-196 P*_hyperspank_-*mKate2 (53) (for experiments in 50 mL of MSgg medium) with genomic DNA originating from the strain B. subtilis NCIB 3610 Δ*epsA–O*::tet (27) and selected for antibiotic resistance to tetracycline (Tet) at 10 μg/mL. Strains were routinely grown in LB medium. MSgg medium was used for biofilm growth and was prepared by combining 20.93 g morpholinepropanesulfonic acid (MOPS), 1.06 g K_3_PO_4_, 0.05 g tryptophan, 0.05 g phenylalanine, 0.41 g MgCl_2_·6H_2_O, 5 g Na-glutamate, and 5 g glycerol, supplemented with 1 mL of microelements (700 μM CaCl_2_, 50 μM FeCl_3_, 50 μM MnCl_2_, and 1 μM ZnCl_2_) per 1 L of distilled water (dH_2_O). pH value was adjusted to 7. After autoclaving, just before performing an experiment, thiamine hydrochloride was added to a final concentration of 2 μM (24). LB medium for agar plates was prepared by adding 35 g of LB agar (Lennox) per 1 L of dH_2_O, solidified through the addition of agar (Sigma-Aldrich) to 2%. The plates were allowed to dry at room temperature overnight.

### Inoculum preparation.

Inoculum for biofilm competition assays for each strain was prepared from spores revitalized in LB for 2 h (shaking at 200 rpm at 37°C) until they reached an optical density (OD) of around 0.2. Two strains were inoculated at a 1:1 ratio (10^5^ spores/mL each) into 2 mL of MSgg medium. Initial colony count (CFU) assays were performed immediately after inoculation on LB agar plates containing spectinomycin (100 μg/mL) for strains labeled with YFP or chloramphenicol (5 for μg/mL) for strains with the mKate2 fluorescence marker. Alternatively, inoculum for biofilm formation, additional competition assays, and testing the incorporation of the Δ*epsA–O* mutant into the pellicle was prepared from cells in exponential growth phase. Briefly, the overnight cultures were inoculated from a colony streaked from −80°C frozen stock culture of each strain and grown in 3 mL of LB medium with spectinomycin (100 μg/mL) or chloramphenicol (5 for μg/mL) for WT strains and spectinomycin (100 μg/mL) or chloramphenicol (5 for μg/mL) and tetracycline (10 μg/mL) for Δ*epsA–O* mutants. After 16 to 18 h of incubation (shaking at 200 rpm at 37°C), 1% inoculum was added to 3 mL of fresh LB medium with the same antibiotics as those used in overnight cultures. Shaken cultures were grown for 3 h to mid-log phase to reach an OD value of around 0.4. This ensured comparable physiological states of each strain used as an inoculum to set up the mixed culture experiments of two strains. OD was measured and adjusted to 0.2, two strains were then mixed at a 1:1 ratio, and 20 μL was inoculated into 2 mL of MSgg medium in a 12-well plate.

### Floating biofilm formation, competition, and testing the incorporation of the Δ*epsA–O* mutant into the floating biofilm when mixed with the wild type.

For monitoring the effect of *epsA–O* mutation on pellicle formation, monoculture biofilms were grown in 2 mL of MSgg as static cultures, which promoted the formation of pellicles at the A-L interphase, in a 12-well plate at 37°C. The pellicles formed after 20 h were photographed and visualized at ×8 magnification using a stereomicroscope (CH9435, type DFC425 C; Leica Microsystems, Wetzlar, Germany).

For counting cells (CFU), mixed pellicles were incubated for 24 h and harvested by coiling on pipette tips, transferred to 1 mL of sterile physiological saline, and disintegrated with an ultrasound sonicator (MSE 150-watt ultrasonic disintegrator Mk2) as described previously (55, 56) (3 times for 5 s with 10 s of pause between each cycle at 20 kHz with an amplitude of 15 μm) to obtain single cells for CFU determination. Sonicated pellicles were serially diluted (by 10-fold steps) in physiological saline and plated on LB agar plates containing the same antibiotics as for initial CFU determination.

### Microscopy.

To examine the spatial distribution of two strains, we again used B. subtilis strains labeled with constitutively expressed YFP or mKate2. Mixed cultures were prepared from revitalized spores inoculated in 2 mL of MSgg medium in 12-well plates as described above (see “Inoculum preparation”). After 16 h of incubation at 37°C, before sporulation started, the medium below the pellicle was removed and pellicles were subjected to CLSM in an AxioVision Z1, LSM800 (Zeiss, Germany). The 16-h time point was chosen because at that time sporulation has not yet been initiated. The CLSM images were acquired by an EC Plan-Neofluar 10×/0.30 Ph 1 objective by two laser channels: the 488-nm laser to acquire fluorescence from the YFP and the 561-nm laser to acquire fluorescence from the mKate2 protein. The emitted fluorescence was recorded at 400 to 600 nm and 600 to 700 nm for YFP and mKate2, respectively. The pinhole size for YFP was set to 1.0 AU, and for mKate2 it was set to 1.2 AU. Frame time was 3.7 s, averaging 4 s, and pixel single-frame size was 930 by 930. The mosaic function of Zen 2.3 (Zeiss, Germany) was used for 2-by-2 frame acquisition. The stitched image of 1,960 by 1,960 pixels covered a field of view with dimensions of 1.2 mm by 1.2 mm. Slicing was set to half of the Nyquist distance, which was 6 μm.

To improve the quality and resolution, the CLSM images were deconvolved by the Tikhonov-Miller algorithm applied in DeconvolutionLab2 application (57) and with artificial point spread function (PSF generator application) (58), both running in a Fiji-ImageJ environment (59). Images were then manually thresholded and converted into binary format, which served as an input for MSSA using the recently developed ImageJ tool (for details, see reference 33).

### MSSA of CLSM biofilm images.

Multiscale spatial segregation analysis (MSSA) determines the segregation level among two strains as a function of the size of the space (field of view of dimensions *d* by *d*) that two strains cooccupy in a biofilm (detailed information available in reference 33). The *d*_min_ corresponded to two times the Nyquist distance (12 μm), and *d*_max_ corresponded to 1.2 mm. The field of view of different *d* is randomly placed on an image, and the strain abundance is defined as an area occupied by the particular strain in the specific field of view. We calculated the local segregation level, *S_d_* (i.e., the segregation in the specific field of view) as

(1) $$S_{d}=\frac{\left|a/A-b/B\right|}{a/A+b/B}$$

where *a* and *b* are abundances of two strains in a specific field of view and *A* and *B* are abundances of two strains in the entire image, i.e., in the field of view with *d*_max_.

By weight averaging many local segregation levels and changing the dimension *d* of field of view from *d*_min_ to *d*_max_, we determined the segregation level, $\hat{S_{d}}$, as a function of *d*. The weights are proportional to the total normalized abundances of the two strains in a particular field of view, (*a*/*A+b*/*B*). In this way the contribution of the empty fields of view to the $\hat{S_{d}}$ is omitted. The segregation level ranges from 0 to 1, where 0 represents no segregation (two differentially labeled strains are simultaneously present in the same field of view in expected amounts) and 1 represents ideal segregation (two differentially labeled cells are never found simultaneously in the examined field of view). The exact values of two extremes of segregation levels, denoted as $\hat{S_{d}^{\text{max}}}$ and $\hat{S_{d}^{\text{min}}}$, are, however, dependent on a particular image and experimental conditions and can be simulated by our approach (33). The $\hat{S_{d}^{\text{max}}}$ and $\hat{S_{d}^{\text{min}}}$ represent positive and negative controls, respectively. To take them into account, one can calculate the relative multiscale spatial segregation level (rMSSL), which is $\hat{S_{d}}$(adjusted for $\hat{S_{d}^{\text{max}}}$ and $\hat{S_{d}^{\text{min}}}$) averaged over all fields of view. This is done by first averaging $\hat{S_{d}}$, $\hat{S_{d}^{\text{max}}}$, and $\hat{S_{d}^{\text{min}}}$ over all field of view dimensions to obtain MSSL, MSSL_max_, and MSSL_min._ (33). The difference of MSSL with MSSL_min_ normalized on the difference of MSSL_max_ with MSSL_min_ represents relative multiscale spatial segregation level (rMSSL). In our software, *MSSegregation* (33), the sampling factor was typically set to 0.005, which means that around 10,000 randomly placed fields of view were evaluated for *S_d_* at *d*_min_. The standard error of the segregation level was in all dimensions of field of view <2%. The resolution factor, which determines the spacing among field of view dimensions for which segregation level will be determined, was set to 0.75. This means that from *d*_max_ to *d*_min_ of field of view we have 22 points on the graph (Fig. 3A), which represents the segregation level versus dimension (*d*) of field of view (distances between points from *d*_max_ to *d*_min_ decrease by the factor 0.75). To simulate the minimal segregation, the bacterial cell was assumed to be a circle of 1.8 μm in diameter, which corresponded well to sizes determined in sonicated samples, where we could resolve the size of the individual cells. During simulations the cells were randomly distributed in a biofilm of the same shape as experimental biofilms, and overlap between cells was allowed. For the calculation of the maximal segregation level, it was assumed that one strain completely excluded the other strain and that thus only this strain is present in each image slice. Also, it was further assumed that the shape of the original biofilm is preserved.

For easier interpretation, it is sometimes useful to convert segregation level, *S_d_* in equation 1, to the ratio in the abundance of the two strains in the field of view by

(2) $$a/b=\frac{\left(1+S_{d}\right)}{\left(1-S_{d}\right)}A/B$$

with *a/A* ≥ *b/B*. This equation also shows that the ratio in the abundance of the two strains in a particular field of view (*a*/*b*) is not a simple replacement for segregation level, because it is dependent on overall abundance of the two strains (*A*/*B*). Therefore, when two different segregation levels (for example, kin versus nonkin) expressed in a ratio of *a*/*b* are compared, the fair comparison can be made only by comparing the two ratios of *a*/*b* at the same overall abundance of the two strains *A*/*B*, for example, by assuming *A*/*B* = 1:1. In such case, when *a*/*b* is >1, it means that one of the two strains will be more abundant in the field of view.

### Statistical analysis.

All experiments were performed in at least three biological replicates in at least two independently repeated experiments. Relative CFU frequencies in the formed pellicles were calculated from bacterial CFU counts [relative CFU frequency of strain A = CFU_A_/(CFU_A_ + CFU_B_)] and are expressed as average frequency values ± standard deviations (SD). Statistical significance of differences between relative frequencies of different strain combinations was determined using a two-sample Student *t* test. *P* values of less than 0.05 were considered significant. The final mean ratios between the Δ*epsA–O* mutant and WT were calculated from bacterial CFU counts and expressed as log_10_ values of the final mean ratio ± SD.
