# Supplementary material for: Kin Discrimination Modifies Strain Distribution, Spatial Segregation, and Incorporation of Extracellular Matrix Polysaccharide Mutants of Bacillus subtilis Strains into Mixed Floating Biofilms
Source: Appl Environ Microbiol. 2022 Sep 12;88(18):e00871-22. doi: 10.1128/aem.00871-22 (PMC9499035; doi:10.1128/aem.00871-22)
Supplement: Supplemental file 1 — Supplemental material. Download aem.00871-22-s0001.pdf, PDF file, 0.9 MB [file aem.00871-22-s0001.pdf]

**Supplementary materials**

# Kin discrimination modifies strain distribution, spatial segregation and incorporation of extracellular matrix polysaccharide mutants of *Bacillus subtilis* strains into mixed floating biofilms

Maja Bolješić[1], Barbara Kraigher[1], Iztok Dogša[1], Barbara Jerič Kokelj[1], Ines Mandić Mulec[1,2*]

[1] University of Ljubljana, Biotechnical Faculty, Department of microbiology, Večna pot 111, SI-1000 Ljubljana, Slovenia

[2] University of Ljubljana, Chair of Microprocess Engineering and Technology – COMPETE, Večna pot 113, SI-1000 Ljubljana, Slovenia

## MATERIALS AND METHODS

### Measurement of floating biofilms growth curves

To measure the floating biofilm growth curve of each strain, inocula were prepared from revitalized spores as described previously (see section **Inocula preparation**). Strains were inoculated as monocultures in 2.45 ml of MSgg in 12-wells plate and incubated for 30 hours at 37ºC in microplate reader (BioTek, Cytation 3 imaging reader) that measured $O.D._{650}$ values of growing pellicles at every hour of incubation.

**Inocula preparation for experiments testing the effect of oxygen limitation on incorporation of the Δ*epsA-O* mutant into the pellicle of the extracellular matrix producer**

The incorporation of the Δ*epsA-O* mutant into the pellicle with EpsA-O-producer in selective oxygen-depleted conditions was tested in 50 ml of MSgg in falcon tubes with loosened lids. Overnight cultures were inoculated from a colony of each strain streaked from -80°C frozen stock culture and grown in 5 ml of LB medium with appropriate antibiotics (shaking at 200 rpm at 37℃). After 16 – 18 hours of incubation cultures were 100x diluted in 5 ml of fresh LB medium with the same antibiotics and grown for 2.5 h in shaking conditions to mid-log phase and then re-inoculated for additional 2.5 hours to reach and O.D. value of around 0.2. Next, two strains were mixed at a 1:1 ratio and 250 µl of each strain was inoculated into 50 ml of MSgg medium.

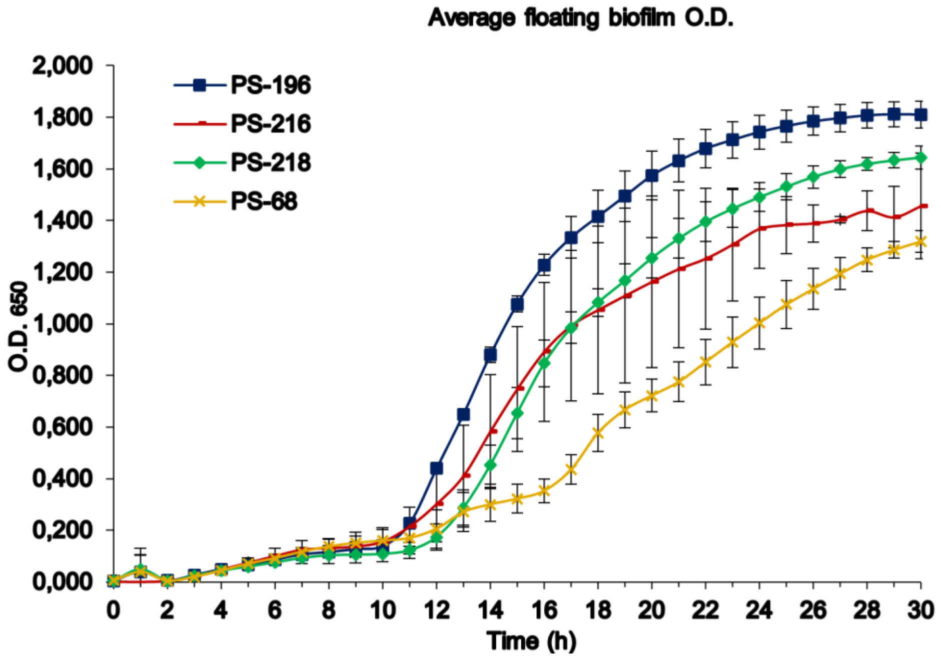

**FIG S1** The floating biofilms growth curves. Selected *B. subtilis* WT strains were inoculated as monocultures in 2.45 ml of MSgg and incubated for 30 hours at 37ºC in microplate reader. O.D. values were measured at every hour of incubation. Experiments were performed in three biological replicates (n = 3).

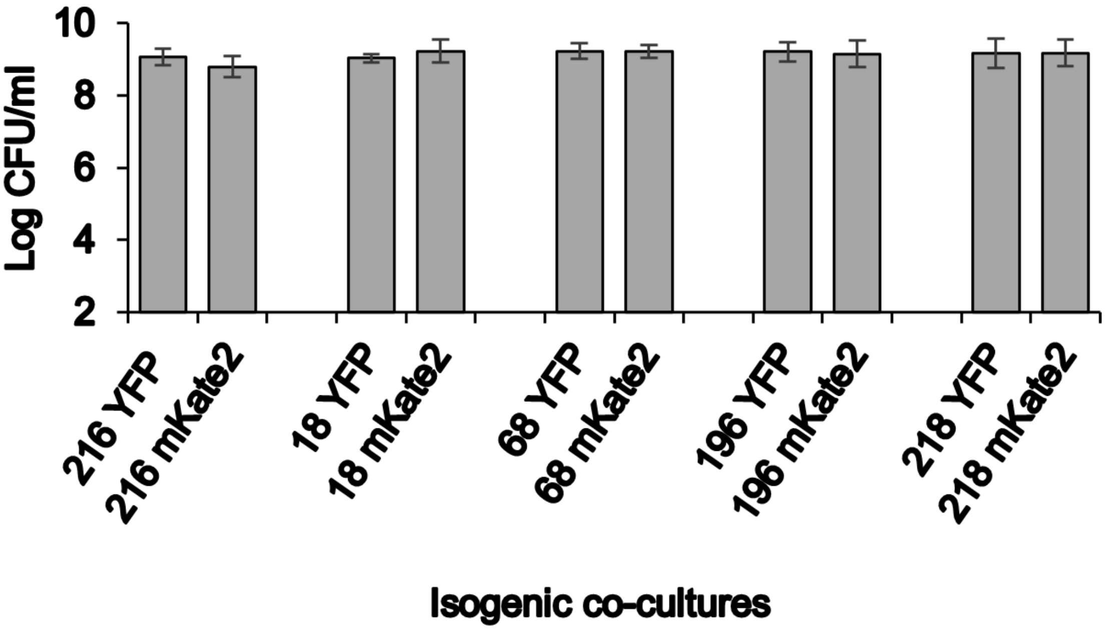


**FIG S2** Cell numbers of isogenic strains in mixed pellicles. *B. subtilis* strains expressing YFP were mixed as revitalized spores with their isogenic strain labeled with mKate2 (at 1:1 ratio) in 2 ml of MSgg and the cell number of each strain was assessed after 24 hours at 37ºC in co-culture pellicles by CFU counts after pellicle sonication. Mixing of two isogenic strains did not result in a significant CFU reduction of either strain in the mixture (*t*-Student; two-tail, $P > 0.05$), which indicates that the type of fluorescent reporter did not affect the fitness of the strain. Moreover, the number of cells in the pellicles did not significantly differ between different tested strains (*t*-Student; two-tail, $P > 0.05$), which

suggests similar fitness in floating biofilms of all tested strains. Experiments were performed in three biological replicates (n = 3).

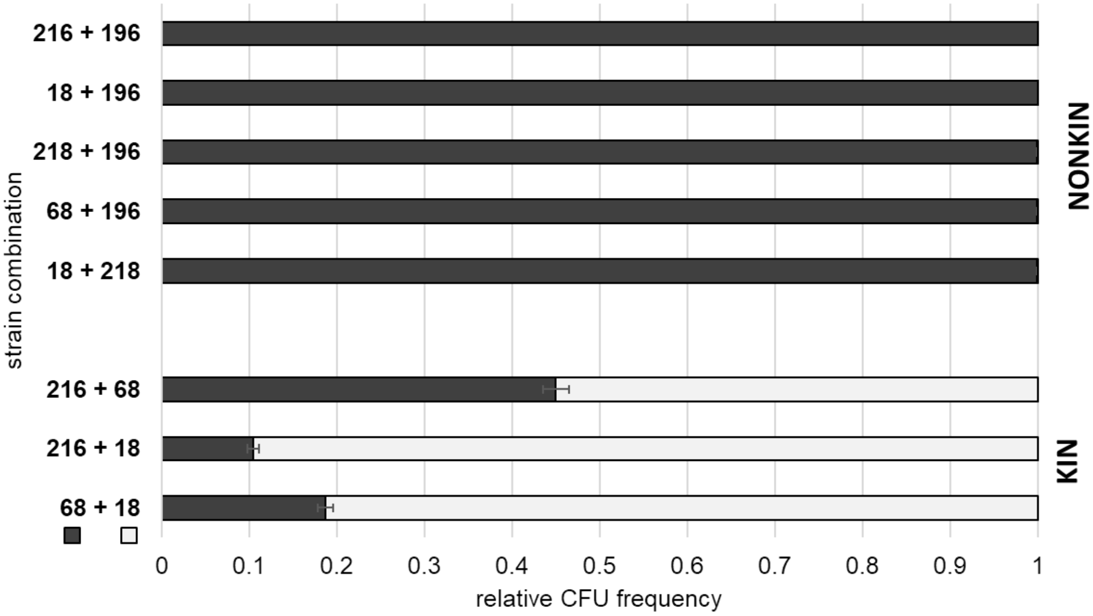


**FIG S3** Relative cell frequency of two strains in mixed pellicles is influenced by nonkin interactions and the exclusion between nonkin is stronger when cells in the exponential growth phase are used as inoculum mix as compared to experiments with revitalized spores (**Fig. 1**). The mean relative cell frequencies (± SD) in the pellicles of three kin and five nonkin strain combinations are shown. Dark grey columns always represent the first strain in the combination. Two strains differentially labeled with different antibiotic resistance markers were mixed in exponential growth phase in given combinations (designated as numbers of PS-strains). They were grown in 2 ml of MSgg for 24 hours at 37°C and the relative frequency of each strain was assessed in the formed pellicles after sonication by CFU counts. CFU counts were performed in two replicate pellicles for strain

combinations PS-216 + PS-18, PS-216 + PS-68 and PS-216 + PS-196 and in four replicate pellicles (in two repeated experiments) for the other strain combinations.

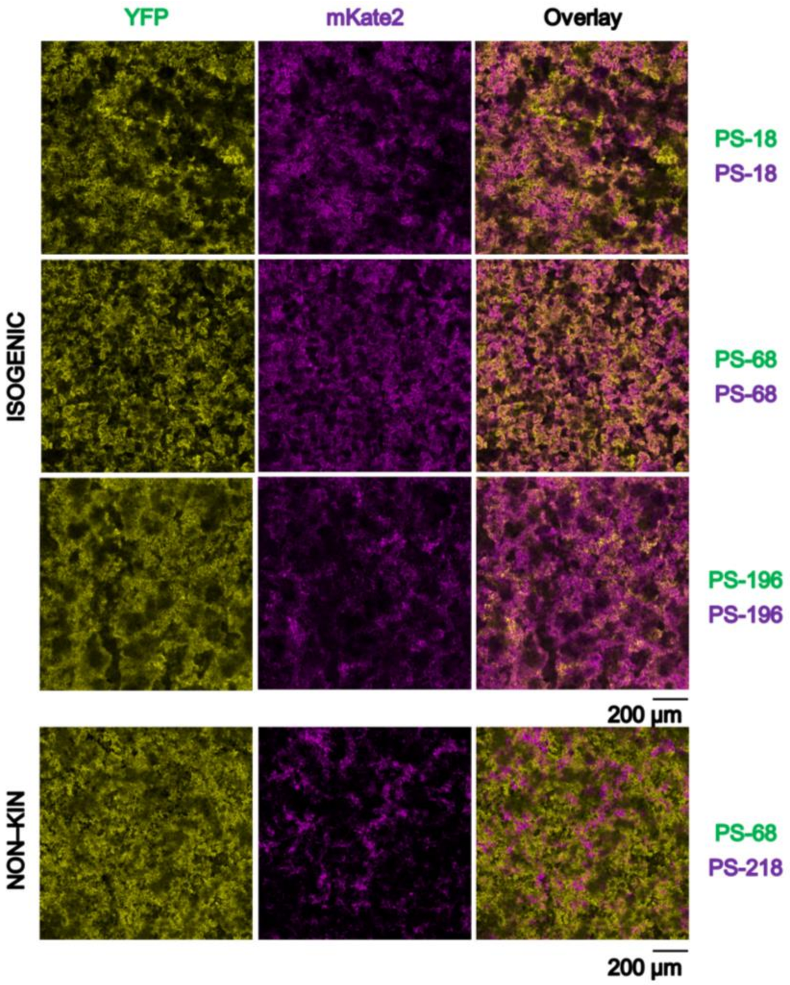


**FIG S4** Fluorescent microscopy images of pellicles composed of two differentially labeled strains in isogenic and nonkin mixtures. Isogenic strain pairs formed biofilms with similar amounts of both co-cultivated strains, whereas in nonkin combination, one genotype prevailed (scale bar, 200 µm).

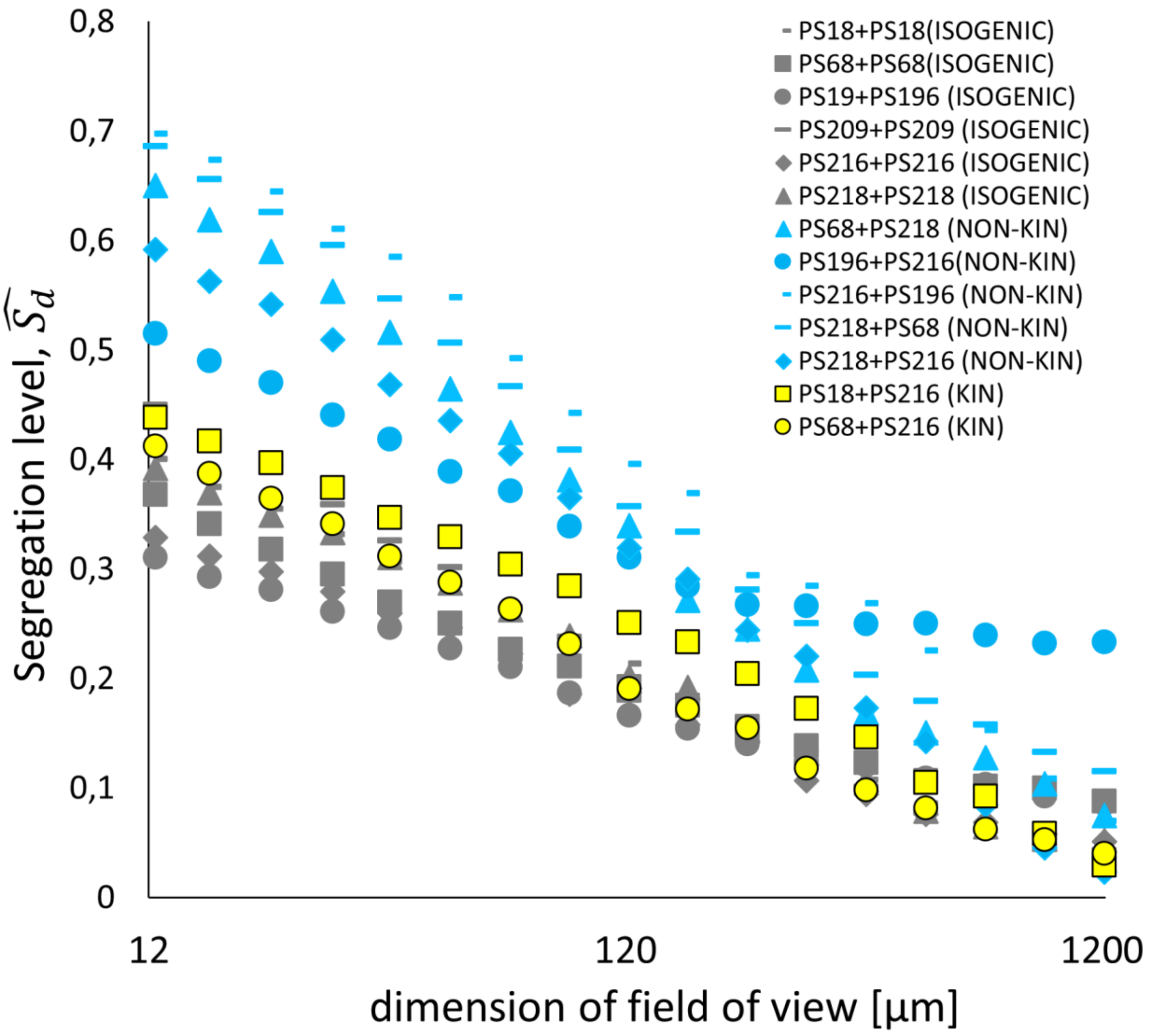


**FIG S5** The plot of segregation levels, $\widehat{S_d}$, calculated by multiscale spatial segregation analysis (MSSA) for mixed floating biofilms of two *B. subtilis* PS strains. The kin (yellow) and isogenic (grey) strain pairs display less segregation compared to nonkin (blue). The negative and positive control curves ( $\widehat{S_d^{max}}$ and $\widehat{S_d^{min}}$ ) are approximately the same in all sample cases (not shown for clarity).

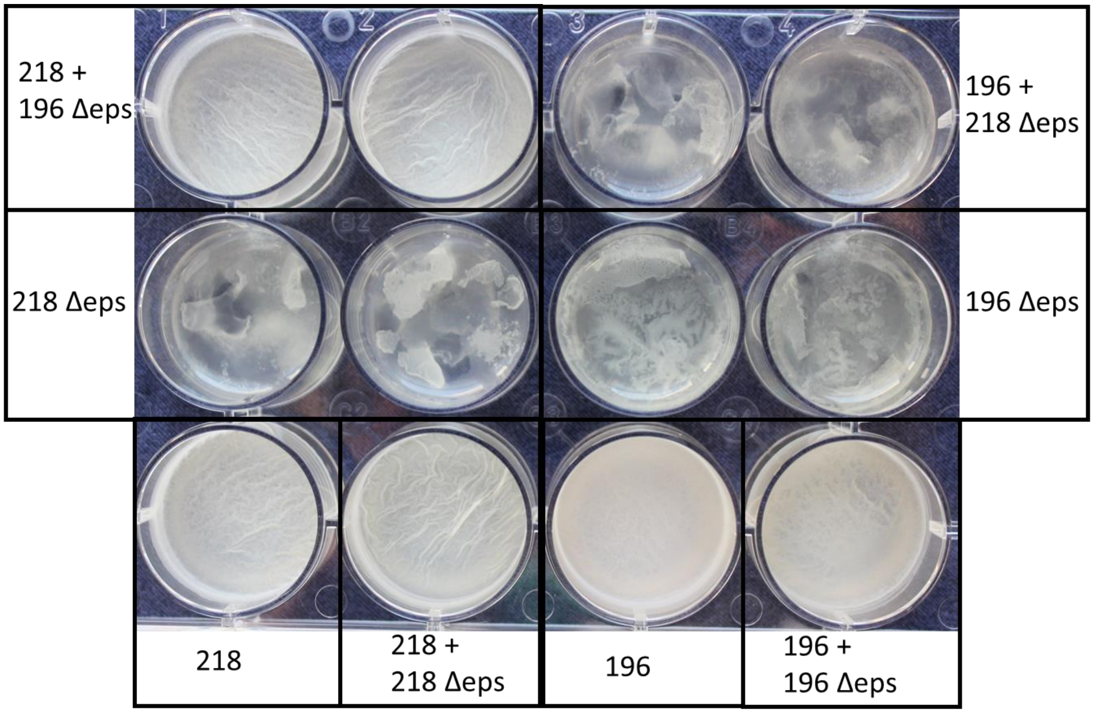


**FIG S6** The dominance in nonkin strain combinations is not EpsA-O production-dependent. The dominant strain PS-218 mixed with PS-196 outcompetes the PS-196 regardless of its deficiency in EpsA-O production, which is evidenced by the absence of firm floating biofilm formation in the mixture of PS-218 Δ*epsA-O* and PS-196. Indicated strain mixtures (or monocultures) were inoculated in exponential phase in 12-well plates in MSgg medium and incubated at 37ºC. Images of the formed biofilms in the wells (diameter 20.5 mm) after 24 hours of growth are shown.

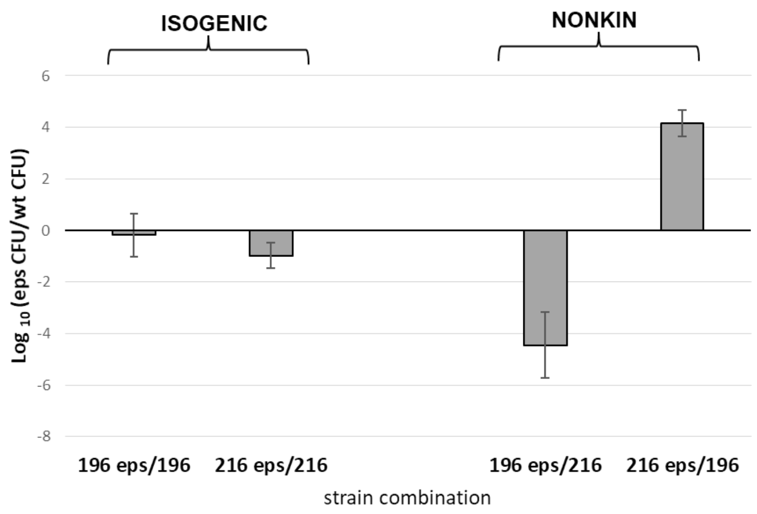


**FIG S7** Incorporation of isogenic Δ*epsA-O* mutant strain into the floating biofilm by the wild-type (WT) EpsA-O-producer and exclusion of nonkin strains in selective oxygen-depleted conditions. Two strains differentially labeled with different antibiotic resistance markers were mixed in exponential growth phase in given combinations (designated as numbers of PS-strains or mutants with eps). They were grown in 50 ml of MSgg in falcon tubes with loosened lids for 24 hours at 37°C. $Log_{10}$-values of the final mean ratio between Δ*epsA-O* mutants and WTs (±SD) determined in the formed pellicles after sonication by CFU counts are shown. Experiments were performed in four biological replicates.

**TABLE S1 STRAINS USED IN THIS STUDY**

| STRAIN | Background | Genome description | Reference |
|---|---|---|---|
| **ZK4101** | NCIB 3610 | *amyE*::P$_{hyperclo3}$-*yfp* (Sp) | (1) |
| **ZK5156** | NCIB 3610 CY49 | *amyE*::P$_{hyperspank}$-*mKate2* (Cm) | (2) |
| **BM1207** | NCIB 3610 | Δ*epsA-O*::tet | (3) |
| **BM1070** | PS-216 | Δ*epsA-O*::tet | (6) |
| **BM1090** | PS-216 | *amyE*::P$_{hyperclo3}$-*yfp* (Sp) | (4) |
| **BM1097** | PS-216 | *amyE*::P$_{hyperspank}$-*mKate2* (Cm) | (5) |
| **BM1313** | PS-196 | *amyE*::P$_{hyperclo3}$-*yfp* (Sp) | (4) |
| **BM1094** | PS-196 | *amyE*::P$_{hyperspank}$-*mKate2* (Cm) | (4) |
| **BM1125** | PS-218 | *amyE*::P$_{hyperclo3}$-*yfp* (Sp) | (4) |
| **BM1098** | PS-218 | *amyE*::P$_{hyperspank}$-*mKate2* (Cm) | (5) |
| **BM1477** | PS-68 | *amyE*::P$_{hyperclo3}$-*yfp* (Sp) | (4) |
| **BM1451** | PS-68 | *amyE*::P$_{hyperspank}$-*mKate2* (Cm) | (4) |
| **BM1516** | PS-18 | *amyE*::P$_{hyperclo3}$-*yfp* (Sp) | (4) |
| **BM1449** | PS-18 | *amyE*::P$_{hyperspank}$-*mKate2* (Cm) | (4) |
| **BM1115** | PS-216 | *amyE::*P$_{tapA}$-*yfp* (Sp) | (5) |
| **BM1113** | PS-196 | *amyE::*P$_{tapA}$-*yfp* (Sp) | (5) |
| **BM1116** | PS-218 | *amyE::*P$_{tapA}$-*yfp* (Sp) | (5) |
| **BM1110** | PS-18 | *amyE::*P$_{tapA}$-*yfp* (Sp) | (5) |
| **BM1597** | PS-216 | Δ*epsA-O*::tet *amyE::*P$_{tapA}$-*yfp* (Sp) | this work |
| **BM1606** | PS-196 | Δ*epsA-O*::tet *amyE::*P$_{tapA}$-*yfp* (Sp) | this work |
| **BM1599** | PS-218 | Δ*epsA-O*::tet *amyE::*P$_{tapA}$-*yfp* (Sp) | this work |
| **BM1601** | PS-18 | Δ*epsA-O*::tet *amyE::*P$_{tapA}$-*yfp* (Sp) | this work |
| **BM1310** | PS-216 | Δ*epsA-O*::tet *amyE::*P$_{hyperspank}$-*mKate2* (Cm) | this work |

| | | | |
|---|---|---|---|
| **BM1306** | PS-196 | Δ*epsA-O*::tet *amyE::*$P_{hyperspank}$-*mKate2* (Cm) | this work |

References


1. Lyons NA, Kraigher B, Stefanic P, Mandic-Mulec I, Kolter R. 2016. A combinatorial kin discrimination system in Bacillus subtilis. Curr Biol 26:733-42.

2. Chen Y, Cao S, Chai Y, Clardy J, Kolter R, Guo JH, Losick R. 2012. A Bacillus subtilis sensor kinase involved in triggering biofilm formation on the roots of tomato plants. Mol Microbiol 85:418-30.

3. Branda SS, Chu F, Kearns DB, Losick R, Kolter R. 2006. A major protein component of the *Bacillus subtilis* biofilm matrix. Mol Microbiol 59:1229-38.

4. Kraigher B, Butolen M, Stefanic P, Mandic Mulec I. 2022. Kin discrimination drives territorial exclusion during *Bacillus subtilis* swarming and restrains exploitation of surfactin. *ISME J* 16**:** 833–841.

5. Stefanic P, Kraigher B, Lyons NA, Kolter R, Mandic-Mulec I. 2015. Kin discrimination between sympatric Bacillus subtilis isolates. Proc Natl Acad Sci U S A 112:14042-7.

6. Stefanic P, Belcijan K, Kraigher B, Kostanjšek R, Nesme J, Madsen J, Kovac J, Sørensen S, Vos M, Mandic-Mulec I. 2021. Kin discrimination promotes horizontal gene transfer between unrelated strains in Bacillus subtilis. Nat Commun 12:3457. https://doi.org/10.1038/s41467-021-23685-w.
